# Tools and methods to study and replicate experiments addressing human social cognition in interactive scenarios

**DOI:** 10.3758/s13428-024-02434-z

**Published:** 2024-05-23

**Authors:** Serena Marchesi, Davide De Tommaso, Kyveli Kompatsiari, Yan Wu, Agnieszka Wykowska

**Affiliations:** 1 Social Cognition in Human-Robot Interaction, Italian Institute of Technology, Genova, Italy; 2 Robotics and Autonomous Systems Department, A*STAR Institute for Infocomm Research, Singapore, Singapore

**Keywords:** Social cognition, Replicability, Interactive scenarios, Humanoid robots

## Abstract

In the last decade, scientists investigating human social cognition have started bringing traditional laboratory paradigms more “into the wild” to examine how socio-cognitive mechanisms of the human brain work in real-life settings. As this implies transferring 2D observational paradigms to 3D interactive environments, there is a risk of compromising experimental control. In this context, we propose a methodological approach which uses humanoid robots as proxies of social interaction partners and embeds them in experimental protocols that adapt classical paradigms of cognitive psychology to interactive scenarios. This allows for a relatively high degree of “naturalness” of interaction and excellent experimental control at the same time. Here, we present two case studies where our methods and tools were applied and replicated across two different laboratories, namely the Italian Institute of Technology in Genova (Italy) and the Agency for Science, Technology and Research in Singapore. In the first case study, we present a replication of an interactive version of a gaze-cueing paradigm reported in Kompatsiari et al. (J Exp Psychol Gen 151(1):121–136, 2022). The second case study presents a replication of a “shared experience” paradigm reported in Marchesi et al. (Technol Mind Behav 3(3):11, 2022). As both studies replicate results across labs and different cultures, we argue that our methods allow for reliable and replicable setups, even though the protocols are complex and involve social interaction. We conclude that our approach can be of benefit to the research field of social cognition and grant higher replicability, for example, in cross-cultural comparisons of social cognition mechanisms.

## Introduction

Traditionally, the study of human social cognition has assumed an “isolated” and “spectatorial” approach (for a review, see Schilbach et al., 2013). This means that most of the research has been conducted with observational paradigms of two-dimensional (2D) stimuli, without real-time interaction (Schurz et al., 2014). Although this approach has led to understanding how we process information about others (Apperly & Butterfill, 2009; Butterfill & Apperly, 2013), a crucial piece of knowledge about human social cognition has been missing—the social interaction. Humans, being intrinsically social, have developed the ability to efficiently predict and interpret others’ behaviors in real time during social interactions (Ebstein et al., 2010; Tomasello et al., 2005). Thus, as argued by Schilbach and colleagues (Schilbach et al., 2013), to truly understand human mechanisms of social cognition, we need to switch our methodological approach from a “static” observational, to an “interactive” approach (i.e. second-person neuroscience) (Bolis & Schilbach, 2020; Caruana et al., 2017; Redcay & Schilbach, 2019). This paradigm shift from 2D stimuli observed in the lab to interactions in the 3D dynamic world have resulted in the study of socio-cognitive mechanisms “in the wild" (for a review see Foulsham et al., 2011; Kingstone et al., 2003; Sebanz et al., 2006).

A more interactive approach to social cognition, however, brought about some challenges. The most evident are the decrease in experimental control and the increase in noise in the data compared with experiments carried out within the “clean” scientific laboratory walls (Holleman et al., 2020). Moreover, often “in-the-wild” studies are not completely reproducible, adding complexity to the interpretation of results that are difficult to replicate (Open Science Collaboration, 2015). As the authors noted in their meta-analysis of reproducibility in psychology, real-world research designs introduce natural variations which laboratory experiments have better control over. This lack of standardization limits direct comparability across similar studies (Baker et al., 2016). The proposed solution seeks to address some of these challenges to reproducibility by establishing clear hypotheses, methodology, and analysis prior to data collection in order to enhance rigor and facilitate future replication efforts. Thus, new methods need to be developed to overcome these issues. One approach is to use virtual reality environments for increasing interactional aspects of the experiments while maintaining experimental control (Pan & Hamilton, 2018). Although this approach is indeed promising, it has one limitation: it does not allow for interaction with the actual physical world or for manipulation of real, physical objects. Therefore, taking this into account, several researchers suggest that using physically embodied artificial agents^1^, such as robots, can be one option to overcome the above challenges and allow for interactions in the real, physical world (Ramsey et al., 2021; Wykowska, 2021). Robots—being endowed with a physical body—can serve as “proxies” of a social interaction partner, allowing for probing human cognitive mechanisms with relatively high “naturalness” of interaction (Wykowska, 2021). Specifically, as humanoid robots are designed to resemble the appearance and movement capabilities of a human body (Dautenhahn, 2007) by having two arms, two legs, a torso, and a head (Fong et al., 2003), they can potentially perform tasks utilizing the same motor repertoire as that of humans. Thanks to such anthropomorphic features, they can be included in interactive protocols, involving joint manipulation of objects or joint action (Ciardo & Wykowska, 2022; Henschel et al., 2020). This makes the robots a closer “proxy” for natural social interaction than virtual characters in a virtual reality setup.

Simultaneously, physically embodied humanoid robots have the advantage of relatively high experimental control compared with interactive protocols involving human confederates or dyads of participants. Thus, they have a higher potential for replicability across labs and various contexts, such as sophisticated tasks involving cooperative work with people in education, elder care, public services, training of cognitive functions, and other collaborative roles (Belpaeme et al., 2018; Ghiglino et al., 2023; Laban et al., 2020, 2022, 2023; Lemaignan et al., 2017). For example, Laban and colleagues (2020; 2022a; 2023) conducted several studies to address whether social robots^2^ could be perceived as conversational partners, to explore their potential also as intervention tools in different social settings. Interestingly, these studies have consistently yielded similar results through various experiments, engaging humans and robots in dyadic social settings. Laban et al. (2020) reported that participants who interact with an embodied artificial social agent are more prone to verbally interact with it, relative to a non-embodied artificial agent; similarly, Laban et al. (2022a) showed that after 5 weeks of interaction, caregivers were engaging longer and with more detail in a conversation with a social robot; while Laban et al. (2023) showed that in a similar length of interaction, participants reported of feeling less lonely and stressed.

It is important to note, however, that although physically embodied robots provide the advantage of making the interaction embedded in the natural physical environment (as opposed to virtual environment provided by virtual reality), they have the disadvantage (compared with virtual reality) of being *robots.* Despite their close physical resemblance to humans (e.g., Ishiguro, 2020), they are still perceived and behave as robots. In contrast, virtual reality allows for designing characters that—although in the virtual (rather than physical) reality—can appear and behave in a more human-like and realistic manner. They also allow for greater flexibility in terms of design of appearance and motor repertoire.

In summary, while various approaches to studying social cognition in a more naturalistic manner offer various advantages and disadvantages (see Table 1), robots allow for studying social cognition in interaction in a natural and physical environment, with high experimental control (Wykowska, 2020, 2021). This approach is of course also not without challenges and has also pros and cons, like other methods. Therefore, the choice of this approach (or other approaches, such as virtual reality) should depend on specific research questions. This represents a new opportunity for the humans’ social cognition field to further investigate it, in parallel with and integrating classical psychological and neuroscientific methodologies. Indeed, robots can be used as tools to investigate human social cognition in diverse contexts and supporting researchers in replicating setups and results (Wykowska, 2020, 2021).

**Table 1:** Comparison of pros and cons across various methodological approaches to study social cognition

		Methodological pros and cons				
		Experimental control	Replicability	Potential generalizability to natural real-life contexts	Ease of use	Cost
	2D stimuli on the screen	Very high	Very high	Low	High	Low
Type of apparatus	Virtual reality	Very high	High	Medium	Low	Medium/high
	Humanoid robots	Very high	High	High	Low	Medium/high
	Human–human interaction	Medium/low	Low	Very high	Medium	Medium

### Examining social cognition with the use of humanoid robots: The case of joint attention

One of the fundamental mechanisms of social cognition is the ability to engage in joint attention. Joint attention occurs when two or more interaction partners attend to the same location or event in the environment (Baron-Cohen et al., 1997). In laboratory settings, joint attention has been operationalized in the form of a gaze-cueing paradigm. In a gaze-cueing paradigm, a face (or a face-like stimulus) is presented on a screen. Typically (but not always in that exact sequence, see (McKay et al., 2021)), first, it is presented with a gaze directed straight ahead (towards the observer). Subsequently, the eyes are presented gazing to a given direction on the screen (often simply left or right). This is the directional “gaze cue” (Friesen & Kingstone, 1998; Galfano et al., 2012; Greene et al., 2009; Hayward & Ristic, 2013). After a certain time interval, defined as stimulus-onset asynchrony (SOA), a target appears at the gazed-at location (validly cued) or other location (invalidly cued). The typical results show better performance (e.g., faster reaction times, RTs) for detection or discrimination of validly cued targets than invalidly cued targets. The difference in RTs between validly and invalidly cued targets reflects a mechanism of attentional orienting (in relation to the directional cue), termed the gaze-cueing effect (GCE). The literature shows that the GCE combines both a bottom-up (i.e., automatic/reflexive) and a top-down (i.e., contextual/social) component (Capozzi & Ristic, 2020; Chevalier et al., 2020; Wiese et al., 2013).

Despite the great corpus of research on GCE, most of the studies are conducted in screen-based settings, with 2D gaze stimuli. However, evidence shows that an embodied gaze contact in a more naturalistic setting can lead to different behavioral and physiological responses relative to a pictorially presented 2D gaze (Chevalier et al., 2020; S. G. Edwards et al., 2015; Hietanen et al., 2008). Thus, recently, Kompatsiari and colleagues used the iCub robot as a “gazer” to provide the gaze cues (Kompatsiari, Bossi, et al., 2021a, 2021b; Kompatsiari, Ciardo, et al., 2018a, 2018b, 2021a, 2021b; Kompatsiari et al., 2022; Kompatsiari, Perez-Osorio, et al., 2018). The authors adapted the gaze-cueing task to a three-dimensional setup, where the robot could establish (or not) mutual gaze with participants. The question of interest was whether eye contact (mutual gaze) would modulate the GCE. First, the authors validated the typical findings of the gaze-cueing paradigm at both the behavioral and neural levels (Kompatsiari, Perez-Osorio, et al., 2018). Next, Kompatsiari, Ciardo et al. (2018a, 2018b; 2022) showed that GCE are modulated by mutual gaze: the GCE was stronger in the mutual gaze condition relative to averted gaze. Furthermore, Kompatsiari and colleagues showed that establishing eye contact with the robot engages human attention, as manifested by longer fixations on iCub’s face during eye contact compared with no eye contact (Kompatsiari, Ciardo, et al., 2021a, 2021b) and modulates humans’ oscillatory brain activity in the same frequency range as in the case of human–human eye contact (alpha frequency) (Kompatsiari, Bossi, et al., 2021a, 2021b).

Taken together, this collection of studies shows that using a human–robot interaction setup to implement a classical paradigm of cognitive psychology that addresses fundamental mechanisms of social cognition not only is feasible and allows for replication of classical effects (GCE) but also allows for gaining new scientific knowledge, namely the impact of eye contact on attention and engagement. Interestingly, while embodied eye contact with a humanoid robot does seem to modulate the GCE, this effect is not observed when the robot face is only presented on the computer screen as a 2D stimulus (Kompatsiari et al., submitted, Marchesi et al., 2023). More specifically, in those studies, a GCE was always observed, independently of the prior gaze type (direct or averted), thereby suggesting that the effect of the more “social” signals, such as eye contact, is more likely to arise in a setting involving the physical presence of an embodied agent, rather than just a pictorial representation on the screen^3^.

### Examining social cognition with the use of humanoid robots: The case of theory of mind

Another fundamental mechanism of social cognition is the theory of mind (Baron-Cohen, 1997). The theory of mind is the ability to reason about others’ mental states and to understand that others’ mental states might be different from one’s own. Scientific literature in the field of developmental psychology and social neuroscience is abundant with results, models, and theories related to the theory of mind (Apperly & Butterfill, 2009; Baron-Cohen et al., 1999). However, here also, the literature is often limited to experimental protocols involving computer screens or vignettes and thus distant from daily-life theory-of-mind situations (Schilbach et al., 2013). Therefore, this area of research also calls for adaptation of classical paradigms to more naturalistic and interactive protocols. One (although not *the only*) approach, as argued above, is to use humanoid robots as proxies of social interaction partners.

It is important to note that before one can translate paradigms addressing theory of mind into human–robot interaction setups, however, one crucial question needs to be asked: does it even make sense to talk about the theory of mind in relation to an artificial agent? Do people attribute mental states to robots? And if so, under which conditions? These questions need to be answered before the theory-of-mind paradigms can be adapted to human–robot interaction protocols.

Recently, several authors have explored how humans interpret the behaviors of a robot and whether they refer to mental states in their explanations and predictions of robot behaviors (Thellman et al., 2022; Thellman & Ziemke, 2020). In a similar vein, Marchesi and colleagues (Marchesi et al., 2019) developed the InStance Test (IST) to probe adoption of the intentional stance towards robots. Intentional stance is a concept introduced by Daniel Dennett (Dennett, 1987) with the idea that humans adopt that stance to explain and predict behaviors of others with reference to mental states. The question of whether humans adopt the intentional stance towards artificial agents has been around for decades in philosophy but had not been operationalized in empirical studies until the work of Thellman and Ziemke (Thellman et al., 2017) or Marchesi et al. (2019)^4^. Marchesi et al.’s test allows one not only to operationalize the philosophical concept of intentional stance (in relation to artificial agents) but also to quantify the degree to which intentional stance is adopted, as the idea is that intentional stance might not be a binary choice but rather a gradient.

The IST of Marchesi et al. (2019) includes 34 scenarios depicting the iCub humanoid robot involved in daily activities. Each scenario is associated with two descriptions: one sentence explains the scenario with the adoption of the design stance, while the other represents the adoption of the intentional stance (using explanations that refer to mental states). In the study by Marchesi et al. (2019), participants were asked to move a cursor along a slider, towards the description that best represented their interpretation of the observed scenario. Results showed that participants adopted, to some extent, the intentional stance towards iCub. This finding led to further exploration of the factors that may influence the adoption of the intentional stance, such as expectations and trust (Perez-Osorio et al., 2019; Vinanzi et al., 2021), behavioral variability and adaptiveness (Ciardo et al., 2022; Vignolo et al., 2022), length of the interaction (Abubshait & Wykowska, 2020), or human likeness in behavior (Bryant et al., 2020; Marchesi et al., 2021, 2022). Human likeness of behavior is quite a critical hint for humans to attribute human traits to artifacts (Ciardo et al., 2022). In the context of intentional stance, Marchesi et al. (2022) developed a paradigm that would engage participants in a daily activity with the robot. More specifically, participants were asked to watch a series of movies together with the iCub robot. In one condition, the robot behaved in a human-like manner (i.e., it reacted to events in the movies in an emotional, relevant, and human-like manner—for example, it laughed at a funny scene or acted as if it was worried in response to a scary scene). In another condition, it would behave very mechanically (i.e., it reacted to events in the movies with beeping sounds of a sensor). In addition, in the human-like condition, the robot interacted with participants before watching the movies. It greeted participants and invited them to the movie-watching session (to access the complete script of the interaction, see https://osf.io/2ckxv). This was achieved through a Wizard-of-Oz (WoOz) manipulation (Rea et al., 2017; Riek, 2012). During this phase, the robot would also make eye contact with the participants via active cameras in its eyes (Kompatsiari, Ciardo, et al., 2018a, 2018b). In the “mechanistic” condition, no eye contact was initiated, and no WoOz manipulation was implemented. Instead of the interaction, the robot displayed “socially detached” behaviors of calibrating motors and preparing cameras to receive input from the screen on which the videos would be played. The results showed that the human-like behavior of the robot increased the likelihood of adopting the intentional stance towards it, as manifested by a higher score in the IST post-interaction relative to the score obtained before the interaction took place. The mechanistic context resulted in no modulation of the likelihood of adopting the intentional stance when scores of IST post-interaction were compared with the scores pre-interaction.

Taken together, this set of results shows that humans are likely to adopt the intentional stance towards humanoid robots to some extent, but interaction with the robot, context and behavioral characteristics of the robot play a role in the degree with which this mechanism occurs. One additional factor that might play a role is cultural embedding.

### The (elusive) role of culture in social cognition

As social signals and social interaction are strongly culturally contextualized (Bandura, 2002; Dalmaso et al., 2022; Hong & Chiu, 2001; Lavelle, 2021, 2022), it is important to consider culture as one of the influential factors impacting social cognition. For example, the amount and duration of eye contact might differ across cultures (Dalmaso et al., 2022; Uono & Hietanen, 2015). Thus, the effect of mutual gaze on attentional orienting might be prone to cultural differences. Similarly, it is also plausible to speculate that adoption of intentional stance towards artificial agents would be modulated by culture in which an individual is embedded (Spatola et al., 2022b).

Indeed, cultural and social norms and values are crucial in the design of social robots because they are meant to interact with humans in social contexts embedded in a given cultural setting (Marchesi & Wykowska, 2023). Social interactions are governed by complex rules and norms that vary across cultures, age groups, and even individual preferences. A social robot that fails to take these factors into account is likely to make mistakes or misunderstandings that can lead to negative experiences for both the robot and the human users. Indeed, Bemelmans and colleagues stress the importance of purposely designing not only the robots, but the intervention contexts, to enhance the efficacy and acceptance of the social robots (Bemelmans et al., 2012). Moreover, in two recent studies, it is reported that humans tend to make causal attribution to robots’ behaviors. In particular, relevant factors that seem to play a pivotal role in how humans perceive social robots seem to be the attributed level of autonomy to the social robots' behaviors (Horstmann & Krämer, 2022) and the corresponding underlying attributed attitudes to these behaviors (A. Edwards & Edwards, 2022). Thus, it is pivotal to consider how the social context (at both the individual and cultural level) affects humans’ perception of social robots.

Despite the increasing number studies that employ various robotic platforms to investigate cross-cultural differences in human–robot interaction (Hong & Chiu, 2001; Lim et al., 2021; Papadopoulos & Koulouglioti, 2018), the role of culture in social cognition is not yet clarified. The different methodologies and the non-homogeneity in replication of the studies have not fully allowed for a clear integration of the cultural factors in the theories related to the mechanisms of social cognition. However, to draw meaningful conclusions about cultural differences in social cognition, one needs to make sure that the paradigms employed are properly replicated across different countries and cultures. This is particularly challenging when one uses interactive naturalistic paradigms. Thinking of human–human interaction studies, it becomes extremely difficult to ensure that the behavior of a confederate in a dyadic interaction would be identical across cultures, given cultural differences in gesticulation, gaze behaviors, and emotional expressivity of the face, for example. On the other hand, perhaps this cultural variability is exactly what is needed to elicit the mechanisms of social cognition across different cultures. This is actually an important empirical theme to examine: how much does social cognition rely on culturally specific behaviors of the interaction partner. This question is difficult to address using human–human interaction protocols, as it is impossible to “turn on/off" cultural variability in gestures, expressiveness, or gaze patterns—something that is entirely doable with robots. Thus, for cross-cultural studies, the use of humanoid robots might also come in handy, though not without challenges. In the two case studies presented in this paper, we decided to use the exact same behaviors of the robot across two different cultures (thus, we did not vary its gestures or expressiveness in a culturally dependent manner), as the focus of this study was to replicate the exact same parameters of the experimental design across labs across continents. Furthermore, the knowledge about culturally specific behaviors, gestures, and expressiveness is rather incomplete, and thus it is not clear how to implement such culturally specific behaviors. Future studies might use culturally specific robot behaviors in a systematic investigation of how such behaviors affect mechanisms of social cognition. However, for this initial step, which aimed to demonstrate replicability of experimental protocols involving a humanoid robot, we opted for keeping robot behaviors constant.

### Challenges in using humanoid robots for the study of social cognition in interaction

Although at the first sight it seems that humanoid robots should, by default, allow for excellent experimental control (and thus replicability across labs and cultures), adaptation of classical psychological paradigms to interactive protocols with humanoid robots is challenging and requires integration of various complex components in the experimental environment, both from the theoretical and from the technical point of view.

From the theoretical perspective, the challenge lies in the fact that interactive protocols often require certain modifications of the classical protocols, which might have theoretical implications. For example, when embedding a gaze-cueing paradigm (as discussed above), one needs to address the issue of stimulus onset asynchrony (SOA). In classical paradigms with face-like stimuli on the screen (Driver et al., 1999; Friesen & Kingstone, 1998), participants are presented with one frame of the gaze directed straight ahead, second frame with gaze directed to a lateral location, and a subsequent frame with target presented laterally. With naturalistic stimuli (e.g., physically embodied head of a robot), the directional cue is provided by a dynamic “stimulus,” namely eyes/head moving continuously to one of the sides (rather than discretely presented frames on the screen). In such case, one needs to decide when the SOA actually starts—is it at the onset of the movement, halfway through the movement, or at the end thereof? The decision of what point during the continuous movement to choose as the beginning of the SOA might then determine the attentional processes involved (e.g., reflexive orienting, which is predominantly observed with short SOAs vs. top-down controlled attentional shifts observed with longer SOAs).

Regarding the technical challenges, they are mainly related to controlling the robot system in an experimental setup integrating various components. Using a humanoid robot for embodying experimental stimuli obviously involves greater complexity than classical screen-based experiments, as it requires a distributed system of computation. From a purely technical point of view, the complexity lies mainly in controlling the physically embodied system in a predictable way. Predictability is a key property of a distributed system in real-time applications since the execution of the distributed processes has predefined critical time constraints. Similarly, experimental protocols with humanoid robots have predefined constraints in time and space that may be violated if the accuracy and the replicability of the stimulation source is not well controlled. Jointly, the integration and synchronization of the input devices used for recording psychophysiological measurements are a critical aspect to consider. Taken together, the technical challenges can be grouped into two main categories, namely, predictability and integration. In this paper, we discuss strategies for ensuring predictability, that is, accuracy and replicability of the stimulation source and methods for integration and synchronization of the stimulation source and the measurement system.

## General methods

This paper presents two case studies, where our solution allowed replication of two exact same experimental protocols across labs and continents. The two validation case studies were related to the key socio-cognitive mechanisms described above: One of the protocols addressed joint attention, operationalized as a gaze-cueing study. The other protocol examined adoption of the intentional stance as a function of human-like behavior of the robot, as discussed in the case study above. We collected data from Italian and Singaporean participants and performed statistical comparisons to examine differences between the two countries. Both studies were first conducted at the Italian Institute of Technology (IIT, Genova, Italy) involving an iCub robot and then were replicated at the Agency for Science, Technology, and Research (A*STAR, Singapore) with another iCub.

### A general framework

In this section, we describe a general methodology to design experimental protocols for studying social cognition in interactive scenarios with robots. The objective is to present experimenters with a framework that facilitates the design of an experimental protocol in cases where a robot is an “interface” to present stimuli to the participant. This framework contains guidelines and good practices that can help speed up prototyping, improve awareness of certain critical issues, and suggest implementation solutions from which to take inspiration.

We propose a four-step methodology that summarizes the main phases to cover and common issues to take into account for the design and development of experimental protocols involving human–robot interaction scenarios.

**Fig. 1:**
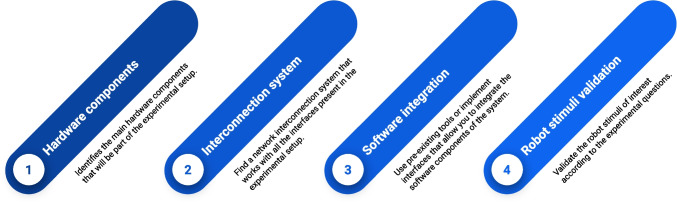
A sequence of four steps to guide experimenters in the design and development of an experimental protocol that uses robots as physical interfaces to interact with

1. **Hardware components** - This step consists of identifying the hardware components involved in the experimental setup. At this stage, we only consider the components of the hardware resources needed to run the experimental protocols, not those in use during the development phase. We have identified as common necessary components, the following: a distributed system consisting of some basic components, such as a main processing machine, input/output interfaces, one or more robotic interfaces, and secondary machines for information processing as shown in Fig. 1. Here, it is important to highlight that some design choices can influence the choice of hardware. Thus, as a best practice to follow, it is critical to think carefully about the need for specific hardware to meet all the experimental requirements.
2. **Interconnection system** - A distributed system is made up of computational units interconnected with each other. Thus, once necessary hardware components are identified, it is necessary to check how they can be connected to each other. A single computer network (e.g., a local area network) might not necessarily be the best choice for all the cases. For example, the presence of high-resolution video streaming (such as those coming from cameras mounted on the robot) could lead to bandwidth overload on the network, negatively contributing to the general latency of all other connected nodes. In this case, the creation of sub-networks in different collision domains is usually a better choice. Thus, in this phase, it is important to decide how best to interconnect the system's hardware components. The suggestion is to identify what is the minimum amount of information to transmit, namely what is the minimum bandwidth required and which nodes are interested in receiving/transmitting this information. Moreover, for time-critical events, a low-latency network connection should always be preferred over the use of wireless connections.
3. **Software integration** - At this stage, it must be ensured that all the necessary solutions are adopted so that the software components can communicate with each other in a reliable manner. This necessitates a reasoning about the operating system, the network protocols, the robotics middleware, and any other software framework and libraries for the use of specific devices.
4. **Robot stimuli validation -** Similar to what would be done in the case of visual stimulation on a screen, stimuli incorporated into a robotic platform will need to be validated. To ensure that the experimental requirements are met in the presentation of the stimuli, in terms of both time and size, in this phase it is important to consider the use of measurement equipment suitable for the specific case.

### The components

**Fig 2:**
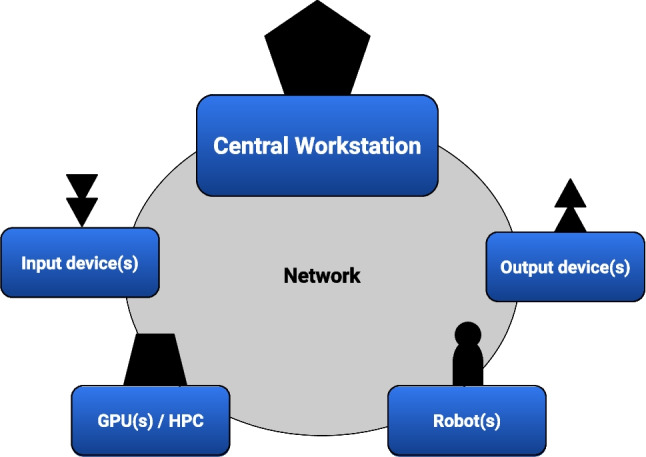
Architecture overview of a general experimental setup involving human–robot interaction scenarios

In general terms, a distributed system (Fig. 2) is needed to run a psychological experiment with a humanoid robot. It consists of a central workstation, input/output device(s), additional processing units (i.e., graphics processing unit(s) or high-performance computing (HPC) systems), and the robotic system(s). We can consider these components as belonging to separate categories even though in practice they might not be. For example, an onboard camera is a part of both the robotic infrastructure and the input interfaces. In the following, these categories of components are described in greater detail.

- *The Central workstation* is a computer in which the experiment code is executed and whose clock is the main time reference for all experimental measurements. It acts as a hub where all devices and peripherals of interest are connected, including those for controlling the robot.
- *The input devices* are all physical interfaces used to collect input from participant (s), such as behavioral responses (i.e., keyboards, touchscreens, touchpads, response boxes, and so on) or psychophysiological measures (i.e., electroencephalography [EEG], electromyography [EMG], eye-tracking devices, and so on).
- *The output devices* are used to present stimuli to the participant(s)—e.g., visual stimuli on screens or audio stimuli using sound cards.
- *GPU or HPC:* Additional processing units are commonly used in real-case scenarios to avoid increasing the computational load of the workstations dealing with high priority controls, such as the robotics controllers or stimuli presentation, and to use dedicated hardware for specific computational tasks required by algorithms for image processing or parallel computing.
- *The robot:* A humanoid robot is a distributed system with electronic boards that control sensors and actuators managed by one or more computational units and, by definition, such electronics are mounted and wired in an anthropomorphically shaped “body.” Given the above categorization, the robot can also be considered as an input and output device. In fact, the sensors can provide measurements while the actuators present stimuli. Both are variables of interest for the experimental study. In this paper, for simplicity, we will treat actuators and sensors as if they were output and input devices, respectively.
- *The software involved*

Typically, a general-purpose operating system is installed in the central workstation (Microsoft Windows, GNU Linux, and MacOS are the most common) where a software for stimulus presentation is installed (i.e., E-Prime, MATLAB, PsychoPy, OpenSesame). Also, depending on how external devices are connected, additional software may be present in the system. In the case of connections through network interfaces, it will be sufficient to interconnect the peripherals in appropriate subnets using network switches. Alternatively, for example with USB devices, specific system libraries or proprietary drivers are required.

#### Addressing the challenge of predictability

One of the first lessons we learned about using humanoid robots in interactive protocols is that we must deal with variability, failures, and delays. The humanoid robots available on the market today are well suited to be controlled for general-purpose tasks, but they lack the reliability and precision of more sophisticated robots designed for real-time applications (i.e., industrial robots, surgical robots).

The issue of predictability is already present in well-controlled experiments even without the use of sophisticated robotic systems. For example, in a screen-based experiment, one must deal with the temporal accuracy in the presentation of visual stimuli due to the refresh rate of the display used. Another typical example is with the use of consumer sound cards, where latencies cause variability between machines and delay the sound delivery time. Having said that, it is easy to understand how the integration of a humanoid robot with higher latencies than a video/audio source can make these temporal inaccuracies more critical to the execution of the experiment. Consequently, the variability in the execution of the robot's actions can produce conditions not always comparable between participants.

**Fig. 3:**
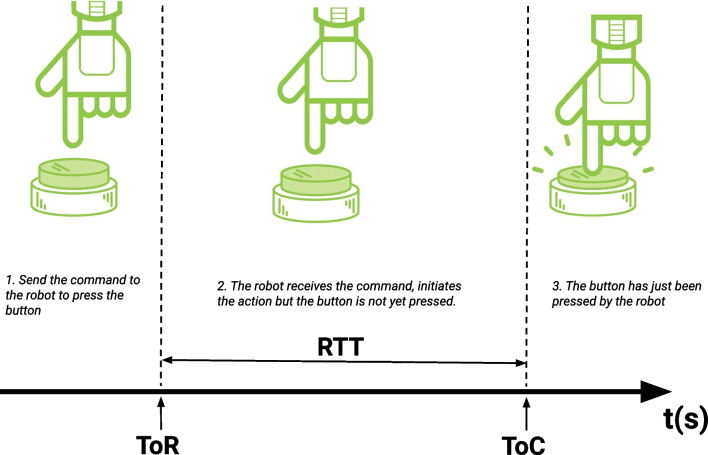
The EOI is represented by the button press of a robotic hand. The three phases show schematically that between the time in which the command is sent to the robot to make an action (a button press in this case) and the time in which this event occurs can vary. Our method suggests the introduction of the RTT metric to measure the latency in the occurrence of the EOI independently of the specific system adopted.

Thus, due to the potential impact that these issues have for the correct execution of the experiment, it is important to find possible strategies to control them. Identifying how long it takes for the robot to process the request made and perform the programmed movement is the first crucial step. To give a quantitative measure of these latencies, we have defined two metrics, namely the event of interest (EOI) and the robot response time (RTT), cf. Fig. 3. First, we need to identify the EOI, namely the stimulus (or action) produced by the robot to which the participant is exposed and whose effect we are interested in studying. At this point, it is important to distinguish between two times, the time in which the stimulus is requested to occur (time of request, ToR) and the time in which the stimulus physically occurs (time of occurrence, ToC). Secondly, we need to compute the RTT associated to that EOI, that is, the time interval between when the associated EOI physically occurs (ToC) and when the request to make an action is sent to the robot (ToR).

We provide some examples inspired by real-case scenarios we have implemented in our research studies. When, for example, the experimental manipulation requires the robot to press a button, we define the “pressing of the button” as the EOI that we want to expose the participant to. In this case, the RRT is calculated as the time interval between when the command is sent to the robot to perform the action and when the button press is received. Thus, the EOI is captured by monitoring the responses of an input device (e.g., keyboard, response box). Another example is when the experimental manipulation requires the robot to look at predefined directions of the workspace. In this case, the RRT is calculated as the time interval between when the command is sent and when the robot gaze reaches a predefined direction, namely a predefined joint configuration of the robot head. In this case, the EOI can be detected by monitoring the encoders of the motors related to the head of the robot. Lastly, when the stimulus to present is to grasp an object, the EOI can be the detection of a specific force value from the sensors of the robot hand.

Thanks to the metric presented here, it should be feasible to measure the value and variability of the robot latency with respect to the EOI. In fact, by repeating the measurements over many trials, it is possible to estimate the distribution of the RRT of interest and what impact it may have on the execution of the experiment. The mean value, the standard deviation, the minimum and maximum values, and other descriptive statistics of the RRT are the measurements of crucial importance to understand the limitations of the robot and, therefore, to readjust the timing of the trial accordingly. Finally, a complete descriptive statistic of RRT is also crucial in the cases where we need to replicate the presentation of the same stimuli in future experiments.

#### Addressing the challenge of integration

For researchers starting to use robots in experimental protocols, the first technical challenge to be addressed is how to integrate these platforms into existing systems. Usually, a general-purpose operating system is installed in the experimental machine where a software for stimuli presentation is installed (e.g., E-Prime, MATLAB, PsychoPy, OpenSesame). This software allows for interfacing with many standard devices, protocols, and proprietary systems. Both commercial and open-source solutions work well with many input and output devices with good time accuracy, but robot systems are not among them yet. This means that the only current viable solution is to write custom implementations. This means integrating the robot controllers in the code of the experiment. This requirement imposes certain criteria for integration: (1) the software for stimuli presentation should allow writing of custom routines or importing custom libraries for controlling the robot, and (2) the network system and protocols need to be compatible with the ones used for controlling the robot. As shown later in the paper in the two case studies, our proposed solution is based on the Python language. While the experiment is developed using the open-source builder OpenSesame (Mathôt et al., 2012), the code for controlling the robot is written in Python as custom routines using Yet Another Robot Platform (YARP) middleware (Metta et al., 2006) bindings. However, there are also hardware integration problems concerning the interconnection between the robotic system and the other hardware components of the experimental setup. These connections refer mainly to those with the central workstation for controlling and accessing the status of the robot. Additionally, other connections can be provided with external measurement devices for triggering robot events, previously defined as EOI. In fact, the measurements collected during the experiments make sense in correspondence with specific events (such as onset/offset of a visual or auditory stimulus). In the case of external units of recording (such as EEG, eye-tracker, transcranial magnetic stimulation [TMS]), the only way to extract the EOIs is by using external triggers. In these scenarios, the integration must provide for specific interconnections between the external recording units and the sensors used to detect the EOI.

#### The use of the iCub robot: A real case study

In our studies, we used the iCub robot^5^ (Metta et al., 2010; Natale et al., 2017). The iCub robot is an open-source platform appreciated and common across various laboratories around the world (Wykowska 2020, 2021). The research and development in robotics and cognitive sciences using this platform are commonly available under open-source licenses and shared among the iCub community (https://icub.iit.it/community/resources). As a result, the availability of hardware and software solutions speeds up the development of new applications, allows for customization, and facilitates bug fixing. Moreover, as a platform located in different research institutions around the globe, it also allows for replicating experimental protocols in research laboratories other than the original ones. The iCub platform also fits well to the requirements of human-like motor repertoire, human-like appearance, and predictability and integration. In fact, the iCub can be controlled well with both MATLAB and Python. These two languages allow the experimenters to easily integrate the robot with well-known software like Psychtoolbox, PsychoPy and OpenSesame. Moreover, the different control strategies enable good quality of movements, having the trajectories of the minimum-jerk profiles, low latencies, and trajectory times with low variability.

### Validation: Two case studies with the iCub humanoid across labs and continents

#### Case Study 1: A gaze-cueing paradigm

The main objective of this validation study was to examine across cultures the GCE that were observed in Kompatsiari et al (2022). Therefore, we implemented in Singapore the exact same paradigm as used by Kompatsiari et al. (2022) in Italy (see Fig. 1). The robot head acted as the gaze-cueing stimulus, directing participants’ attention to one of the laterally positioned screens. Participants’ task was to discriminate the letter T from V, presented on one of the screens. The important manipulation was eye contact with the iCub—in one condition, iCub engaged participants in eye contact at the beginning of each trial; in the other, it averted its gaze from participants’ eyes.

### Methods: Case Study 1

**Fig. 4:**
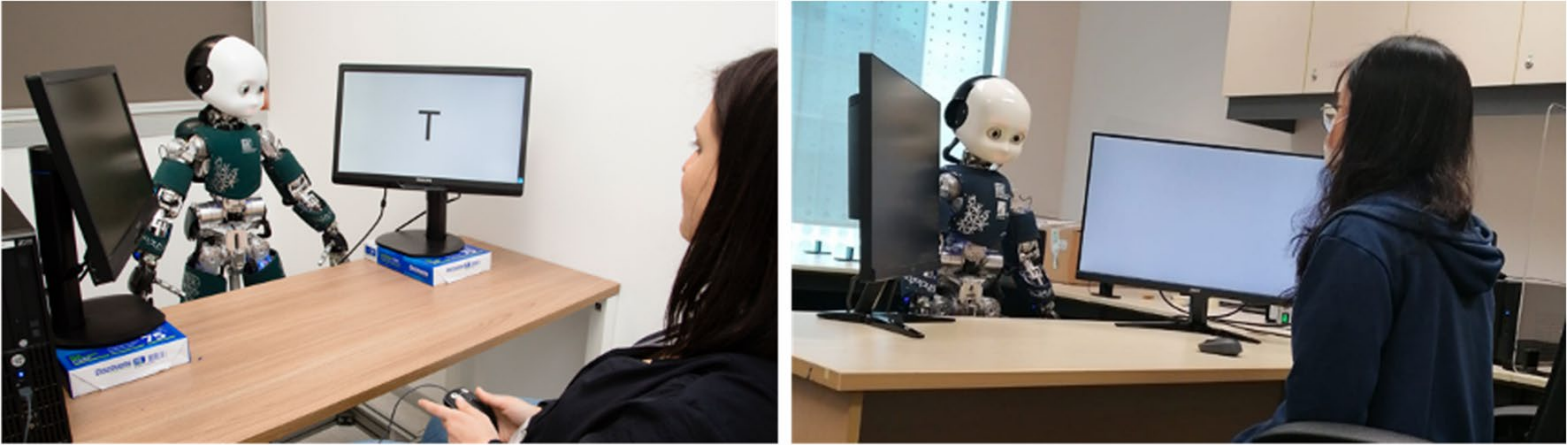
The two experimental gaze-cueing setups in Italy (left) and in Singapore (right)

In the present study, we conducted the experiment with the following procedure: the setup was placed in an isolated and noise-attenuated room. Participants were seated in front of a desk where two 27-inch screens were laterally positioned (75 cm apart, center-to-center) at a viewing distance of 100 cm from the participant’s nose apex, see Fig. 4. The screens were tilted back (by approximately 14° from the vertical position) and were rotated (to the right for the right screen or to the left for the left screen) by approximately 76°. The target stimuli consisted of two letters appearing on either screen (a “V” or a “T,” appearing 3° 32 high, 4° 5′ wide, visual angle). iCub was positioned between the screens, opposite to the participant. Participants’ and iCub’s eyes were at the same height (122 cm from the floor). iCub directed its gaze to one of five possible positions: resting—towards a point in space between the desk and participant’s upper body; eye contact—towards participants’ eyes; no eye contact—towards an empty space on the desk in front of the participant; left—towards the target letter on the left screen; and right—towards the target letter on the right screen (see procedure in Kompatsiari et al. 2018b, Kompatsiari et al., 2019b)

### The active modules to control the iCub robot

**Fig. 5:**
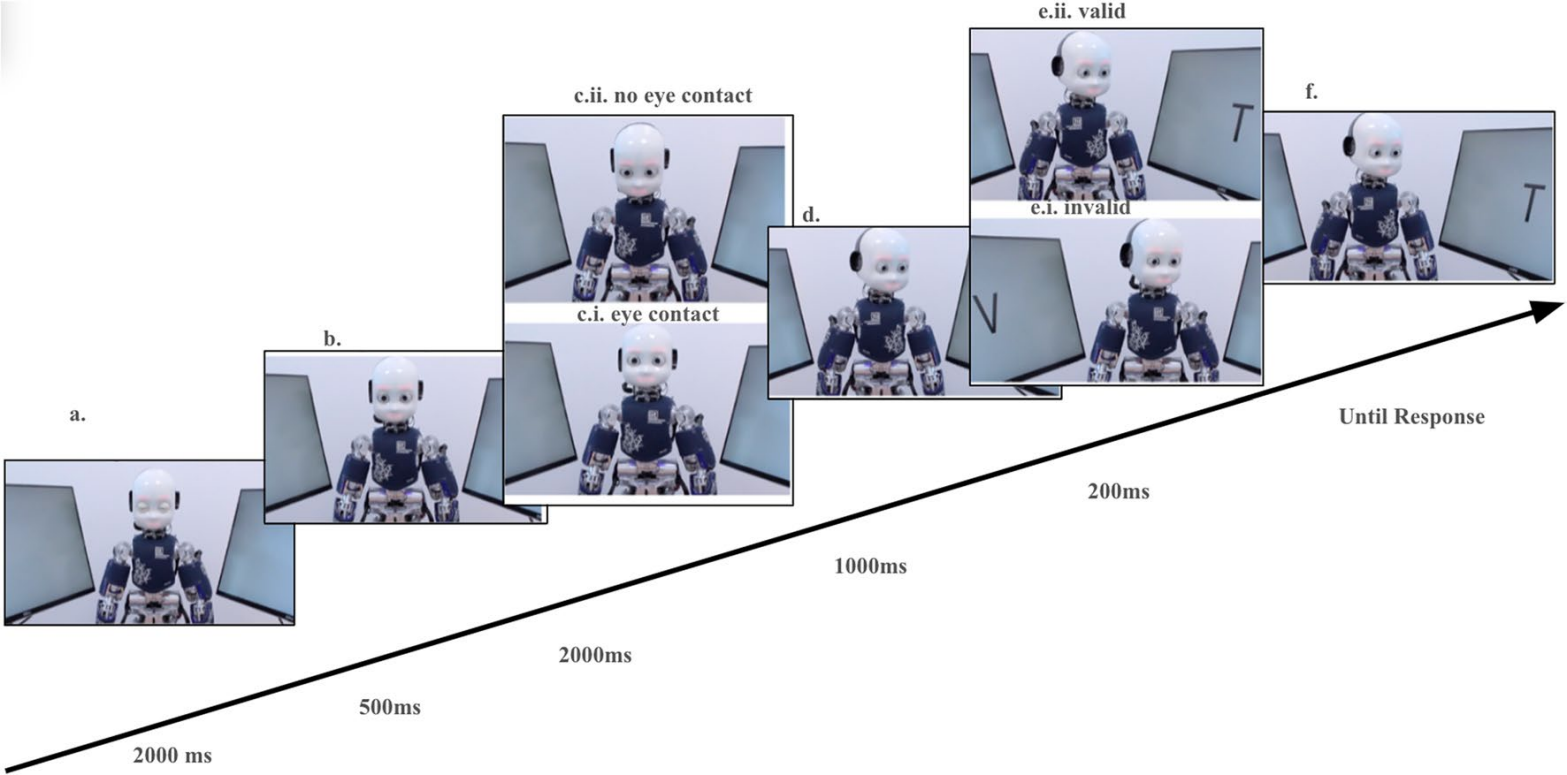
Trial sequence for Experiment 1. (**a**) iCub starts with its eyes closed for 2 s. (**b**) Subsequently, it opens the eyes in the same position. (**c**) After 500 ms, it either establishes eye contact with the participants (Eye contact condition: c.i.) or looks downward (No eye contact condition c.ii) for 2 s. (**d**) Then, iCub looks laterally towards a potential target location. (**e**) 1000 ms after the onset of the robot’s head movement, the target letter (Τ or V) appears for 200 ms. The robot’s gaze is not predictive of the target location (50% validity, Valid condition: e.i., Invalid condition: e.ii.). (**f**) The participant (not shown) discriminates against the target by pressing T or V (Kompatsiari et al., 2022).

To control the iCub’s gaze we used the iKinGazeCtrl controller (Roncone et al., 2016), via the middleware platform YARP. Using this controller, the joints' movements were produced following a minimum-jerk velocity profile. The robot moved its head together with the eyes, allowing a more naturalistic gaze behavior relative to gaze-only movements. The trajectory time for the movement of the eyes and the neck was set to 200 ms and 400 ms, respectively, while the vergence of the eyes was set to 3.5° and maintained constant. Participants’ eyes were detected by the robot’s eyes (stereo cameras) using the face detector algorithm of the [https://github.com/robotology/human-sensing] repository. When the algorithm did not find participants’ eyes, the robot was programmed to look straight at a predefined position in space, allowing the establishment of eye contact with the participant seated in such a way that eyes would be at the same level as iCub’s eyes. iCub’s gaze positions were defined according to the predefined angle values of pitch, roll, and yaw of the neck’s joints. The angles were selected adequately to ensure the same joints’ shift between the eye contact and no eye contact condition Fig. 5.

### Behavioral results from Case Study 1

We collected *N* = 44 Singaporean participants (*F* = 29, mean age = 32.5). Data from nine participants were excluded due to technical issues resulting in an insufficient number of valid trials (onset of the robot head movement exceeded a predefined time window). The final sample was *N* = 35 (*F* = 23, mean age = 31.23). The study was approved by the local ethics committee in Singapore and was conducted in accordance with the Code of Ethics of the World Medical Association (Declaration of Helsinki). Each participant provided written informed consent before taking part in the experiment. All participants were naïve to the purpose of this experiment. All participants received shopping vouchers valued at 30 SGD as compensation for their participation in the study. Data were preprocessed and analyzed using R (version 4.2.0) and JASP (version 0.16.2).

We preprocessed our data first by excluding speed outlier trials (< 150 ms and >1500 ms). After this procedure, trials that were greater than two standard deviations with respect to the individual overall mean averaged across all conditions were considered outliers and, thus, excluded. After these procedures were applied, data from the Singaporean sample were compared with the Italian sample from Kompatsiari et al. (2022) (*N* = 32) by means of a mixed-design analysis of variance (MD-ANOVA). We ran a 2×2×2 MD-ANOVA to investigate participants' reaction times (RTs) during the task and whether the different nationalities could affect the cognitive processes of joint attention involved in the task. Thus, gaze type (avoiding vs. mutual) and validity (valid vs. invalid) were considered as the two within-participant factors, with two levels each. Nationality (Singapore vs. Italy) was considered as between-participant factor, with two levels.

Results revealed a significant main effect of validity, *F*(1,65): 39.5, *p* < .001, η2_p_ = .38, and a significant two-way interaction between validity and gaze type, *F*(1,65): 10.09, *p* = .002, η2_p_ = .13 (see Table 2 for summary of mean RTs per condition). No main effect of nationality *F*(1,65): 3.4, *p* = .07, η2_p_ = .05], or effect of nationality in interaction with gaze type or validity emerged as significant. Post hoc comparisons were performed on the interaction between validity and gaze type with Bonferroni correction and showed that RTs were faster for valid trials than invalid trials for both mutual [*p*_bonf_ < .001] and avoiding condition [*p*_bonf_ = .004]

**Table 2:** Validity effects (valid vs. invalid) as a function of nationality (Italy vs. Singapore) and gaze contact (mutual vs. avoiding)

Gaze_type		Nationality	Validity	Mean	*SD*
Mutual	Italy		Valid	478.85	68.89
	Italy		Invalid	493.01	74.44
	Singapore		Valid	445.49	72.07
	Singapore		Invalid	462.45	79.09
Avoiding	Italy		Valid	480.61	74.51
	Italy		Invalid	485.65	76.20
	Singapore		Valid	444.09	72.05
	Singapore		Invalid	454.70	74.31

### Discussion: Case Study 1

Case Study 1 was designed to replicate a gaze-cueing study reported as Experiment 1 by Kompatsiari et al. (2022), conducted in Italy. Indeed, our findings confirm that mutual gaze elicited stronger GCE than the gaze avoidance condition in both Italian and Singaporean samples. Although the GCE was present in both mutual and gaze avoidance conditions (as indicated by post hoc comparisons), the significant interaction of validity and gaze type suggests that it was stronger in the mutual gaze condition. This pattern of the GCE confirms that the social component can modulate attentional orienting in relation to gaze direction. Interestingly, the lack of any effect (either main or interaction) of nationality suggests that the modulation of these mechanisms elicited by an artificial social agent can be generalized across different labs and cultures.

#### Case Study 2: The intentional stance

**Methods: Case Study 2:** For Case Study 2, we recreated in Singapore the exact setup that Marchesi. et al. (2022) designed in Italy. The aim of that study was to examine whether the likelihood of adopting the intentional stance towards the iCub robot changes after a shared social experience of movie watching with the robot. As described in the original paper (Marchesi et al., 2022), after the completion of the InStance Test (IST, Marchesi et al., 2019), participants were instructed to sit in a room beside the robot (approximately 1.30 m distance) (see Fig. 2). They were told that the task would consist of watching three documentary videos with the robot. Each video was edited to last 1.21 min, for a total duration of 4.3 min. Although Marchesi et al. (2022) report three experiments, since the only difference between Experiments 1 and 2 was concerning the way the items of the IST were split into a pre- and post “half”-test (that is, which items of the entire IST were grouped into pre-test and which were administered post-interaction), and not concerning the setup or the robot’s behaviors. Here, we replicated only Experiments 2 and 3. Experiment 2 presented participants with human-like reactions of the robot to the content of the movie, while in Experiment 3, the robot displayed completely mechanical behaviors (for videos demonstrating the respective behaviors, see: https://osf.io/2ckxv). Furthermore, in Experiment 2, the robot interacted with the participants via a WoOz manipulation (Kelley, 1984; for a review see Riek, 2012) before the video part would start. This type of manipulation allows the experimenter to remotely control completely (or partially) the actions of a robot during an interaction, including movements, speech, gestures, and more (for a detailed review, refer to Riek, 2012). This manipulation is used to achieve natural interaction without relying on artificial intelligence (AI) solutions that would enable the robot to autonomously exhibit similar behavior. Additionally, as part of the WoOz interaction, the robot directly addresses participants, and its cameras, located in its eyes, actively recognize the participants' faces to establish mutual gaze between the iCub and the participants. The WoOz part of the interaction consisted of the following steps:

(i) At the beginning of the video session, the robot would greet participants, introduce itself, ask participants’ names, and invite them to watch some videos together (to access the full script of the interaction see https://osf.io/2ckxv).
(ii) At the end of the video session, the robot would say goodbye to the participants and invite them to proceed to fill out questionnaires.

The human-like behavior of the robot during the video session consisted of iCub showing vocal and facial emotional reactions to the videos. In Experiment 3, any type of social interaction with the robot was removed. Instead of the WoOz interaction, the robot issued verbal utterances about the calibration process it was undergoing. All the emotional sounds presented in Experiments 2 during the videos were replaced with a “beep” sound. In both experiments, all sound and recordings were played via two speakers positioned on the floor behind the robot, creating the impression that the source of the sound was the robot itself. Materials of the original study are available at https://osf.io/xnm5c/ .

**Fig. 6:**
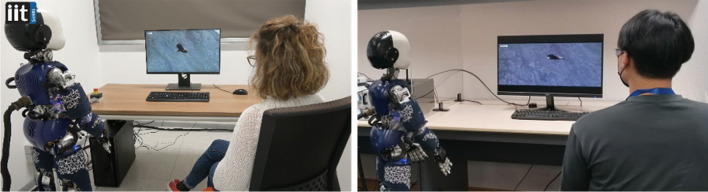
The two experimental “shared experience’ setups in Italy (left) and in Singapore (right)

To summarize, in Case Study 2, participants went through the following steps: Step 1- They completed the IST, assessing their tendency to adopt their intentional stance towards the iCub robot before any interaction (Pre-IST). Step 2- They were seated beside the robot and instructed to watch some movies together. The robot would react to the events in the videos either in a humanlike way (Group1) or in a machinelike way (Group2). Step 3- Finally, their tendency to adopt the intentional stance towards the iCub robot was measured again with the second half of the IST (post-IST). This structure allowed us to measure modulation of the tendency to adopt the intentional stance towards the iCub robot related to the behaviors exerted by the robot in step 2. See Fig. 6 for the experimental setups.

### The active modules to control the iCub robot

In Marchesi et al (2022), the authors designed and validated three different behaviors of the iCub robot (sadness, awe, and happiness). These behaviors were displayed as contingent reactions to the events occurring in the documentary videos presented to iCub and the participants. To implement movements that would be perceived as human-like as possible, the authors followed the principles of animation (Sultana et al., 2013). The implementation was done via the middleware Yet Another Robot Platform (YARP; Metta et al., 2006) using the position controller following a minimum jerk profile for head, torso, and arm joint movements. To implement the gaze behavior, the authors used the 6-DoF iKinGazeCtrl (Roncone et al., 2016) based on inverse kinematics to produce eye and neck movements. All behaviors were programmed to occur at the climax event of each video. Finally, to augment the human likeness during the verbal interaction, the emotional reactions and the utterances were prerecorded by an actor and digitally edited to match the childish appearance of the iCub using the Audacity cross-platform sound editor. The greeting sentences were played by the experimenter via a Wizard-of-Oz manipulation (WoOz; Kelley, 1983). With regard to the mechanistic condition, Marchesi et al. (2022) tailored the robot's responses to the videos so that it consistently executed repetitive movements of the torso, head, and neck. The robot's cameras were switched off, eliminating any possibility of mutual gaze between the robot and the participants. The WoOz manipulation was substituted with preprogrammed robotic actions, such as joint calibration. Verbal interaction was replaced by a verbal description of the robot's calibration sequences, which was generated and presented using text-to-speech technology. Furthermore, all emotional sounds featured in the human-like condition were replaced by a simple "beep" sound.

### Behavioral results from Case Study 2

Following Marchesi et al., (2022), we collected two separate samples of Singaporean participants: one sample interacted with the human-like robot (*N* = 72, *F* = 33, mean age = 35.41) and another sample with the machine-like robot (*N* = 40, *F* = 22, mean age = 43.47). Twenty participants were excluded from the human-like condition (final sample: *N* = 52, F = 26, mean age = 35.5) due to mechanical issues during the experimental session (such as mechanical joints failing or failure of the face recognition due to wearing masks). Since we noticed that many participants were being excluded throughout the experiment, we collected more data sets to ensure matching the minimum *N* required to replicate Marchesi et al. study (*N* = 40), as we did not know whether more participants would need to be excluded during data analysis. However, given that we did not experience any issue while collecting data for the machine-like condition, we stopped data collection at *N* = 40. The study was approved by the local Ethical Committee in Singapore and was conducted in accordance with the Code of Ethics of the World Medical Association (Declaration of Helsinki). Each participant provided written informed consent before taking part in the experiment. All participants were naïve to the purpose of this experiment. All participants received shopping vouchers valued at 20 SGD as compensation for their participation in the study. Data were preprocessed and analyzed using R (version 4.2.0) and JASP (version 0.16.2).

To compare the IST scores between the two experiments run in Singapore with the two experiments run in Italy, we performed a MD-ANOVA where the order of the IST (Pre vs. Post) was considered as a within-subject factor with two levels, and nationality (Italy vs. Singapore) and robot behavior (human-like vs. machine-like) were considered between-subject factors with two levels each. The main effect of IST order emerged as significant, *F*(1,168): 14.37, *p* < .001, η2_p_ = .08, showing that the IST-Post differed from the IST-Pre in general (see Table 3 for a summary of the means and standard deviations of the IST by condition). Moreover, the two-way interaction between IST and robot behavior emerged as significant, *F*(1,168): 12.5, *p* <.001, η2_p_ = .07, showing that the effect of IST order was driven mainly by the human-like condition [Humanlike_Pre vs. Humalike_Post: *p*_bonf_ < .001; Machinelike_Pre vs. Machinelike_Post: p_bonf_ = 1]. Importantly, the contrast between Humanlike_Pre and Machinelike_Pre did not emerge as significant [*p*_bonf_ = 1], indicating that the pre-IST scores (IST scores prior to interaction) were comparable across groups. Finally, no main or interaction effects of nationality emerged as significant (all *p*s >.07).

**Table 3:** Intentional Stance Test score as a function of order (pre- vs. post-interaction), robot behavior (human-like vs. mechanical), and nationality (Singapore vs. Italy)

Nationality	Robot behavior	IST	Mean	*SD*
Italy	Human-like	Pre	41.72	14.28
		Post	54.08	16.65
	Machine-like	Pre	43.41	14.62
		Post	44.97	16.31
Singapore	Human-like	Pre	42.77	14.52
		Post	48.62	21.36
	Machine-like	Pre	43.89	13.89
		Post	43.01	18.65

### Discussion:Case Study 2

Case Study 2 was designed to replicate in Singapore the setup used by Marchesi et al. (2022) in Italy. More specifically, we replicated the experimental setups reported in Experiments 2 and 3 of Marchesi et al. (2022), where two groups of participants were interacting with the iCub showing two different behaviors (one per group): a humanlike behavior (Experiment 2) and a machine-like behavior (Experiment 3). The results revealed that participants showed a higher likelihood of adopting the intentional stance (measured by means of the IST) after interaction with the human-like behaving robot, independently of their nationality. They did not show an increased IST score post-interaction when the robot behaved in a machine-like manner. This was also independent of nationality. Thus, our results show that the effect of human-like behavior of the robot in a joint social activity on adoption of the intentional stance is robust enough to generalize across labs and continents.

## General discussion

In this study, we aimed to demonstrate that the use of a humanoid robot in interactive protocols is a good methodological choice for studying mechanisms of social cognition in 3D protocols that allows for excellent experimental control (unlike designs that take social cognition research “into the wild”). We demonstrated that even though our experimental protocols involved interaction, we could reliably transfer our setup across continents, thereby allowing for cross-cultural studies with replicable setups. Our reliable experimental infrastructure allowed us to replicate the experimental environments from two case studies reported in the literature (Kompatsiari et al., 2022; Marchesi et al., 2022) that addressed fundamental mechanisms of social cognition: joint attention and adoption of intentional stance. We showed comparable results across the two countries: both the GCE effects (+ their modulation by mutual gaze) and the effect of human-like behavior of the robot on the intentional stance score showed the same pattern in Singapore as in Italy. Although this demonstrates a lack of cross-cultural differences in fundamental mechanisms in social cognition, it is an important piece of knowledge regarding replicability of effects that have been obtained with very sophisticated and complex setups. In general terms, we demonstrate that the use of a humanoid robotic platform, such as (but not limited to) the iCub robot, is beneficial for studying mechanisms of social cognition in interaction. That is because it allows for a naturalistic interaction in the study of socio-cognitive mechanisms, while not jeopardizing experimental control. The successful transfer of the same interactive setup between continents with comparable results obtained is a proof of concept that experiments involving an interaction with a humanoid robot—used as a proxy for social interaction partner—can be reproduced in a reliable manner even across continents.

## Future directions

Although the studies presented here have focused only on studying mechanisms of *social* cognition with the humanoid robot, this approach is obviously generalizable to studying also other cognitive and affective domains, such as cognitive control (Spatola et al., 2020, 2022a) or emotions (Rosenthal-von Der Pütten & Bock, 2023). Future studies are encouraged to examine the replicability of the results obtained with the use of human–robot interaction protocols not only in the social domain but also in the context of studying cognitive and affective mechanisms. Furthermore, the high potential for replicability of experimental setups involving a humanoid robot not only can benefit fundamental research performed in the lab but also can extend to applied domains. For example, Ghiglino et al. (2023) developed an experimental environment where the iCub robot was integrated with a training protocol for children diagnosed with autism spectrum disorder (ASD). The authors developed the paradigm and integrated the iCub robot by means of the same solution as described here, first in the laboratories at IIT (Genova, Italy), and later translating the experimental environment on a different iCub robot at a rehabilitation center in Genova to conduct the robot-assisted training. Thanks to this approach, subsequent steps are simply to implement a similar architecture as the one presented in the present paper to allow the clinical trainers and therapists to autonomously run the clinical protocol with the iCub robot, even if they do not have any background in robotics. This shows how our solutions can flexibly adapt to various users and to protocols that range from experimental setups in the lab to clinical intervention (training) in a sensitive environment.

## Footnotes

1 By physically embodied artificial agents we mean entities that have the capacity to act (agents) which are not natural (such as humans or animals) but rather artificial (man-made) and are physically embodied (meaning that have a physical (rather than just virtual) “hardware” in the shape of a body with sensors and effectors)

2 Social robots are defined as robots designed to assist humans in various real-life contexts (childcare, elderly care, healthcare), providing not only service but also social companionship.

3 Of note is that these studies did not directly compare GCE for robot faces relative to human faces, as the focus was to examine whether (and to what extent) a pictorial representation of a robot face could elicit a GCE, and how this compares to the physically embodied version of the paradigm. Similarly, these studies did not focus on the temporal dynamics of the effect, and hence did not analyze the evolution of the effect over the course of the experiment. Thus, the reported GCE are simply an average across the entire experimental session, per condition of interest.

4 Note that we distinguish the concept of intentional stance from the concept of theory of mind (which has been operationalized abundantly in literature). In our view, theory of mind refers to attributing a particular mental state in a particular situation (as in the false belief task in the “Sally and Anne” paradigm [Wimmer and Perner, 1983; Baron-Cohen, Leslie and Frith, 1985], where Anne believes that her toy is in a different basket than where it actually is). On the contrary, intentional stance is the general stance of seeing another agent as being capable of having mental states in general. In consequence, one can fail the theory-of-mind test (attributing wrong mental state to Anne), but this does not mean that they do not attribute mental states to Anne in general. They might still ascribe the intentional stance to Anne while attributing the incorrect specific mental state in a given context.

5 Please note that iCub is not the only humanoid robot that can be used in human–robot interaction studies to examine human social cognition. There are other (commercial) robot platforms that might be considered (e.g., the NAO or Pepper robots from Aldebaran/United Robotics Group or ARI from PAL Robotics). However, the choice of the robot will depend on the specific experimental questions and setup—for example, if the experimental question requires the robot to have a very similar manual motor repertoire to a human, then iCub is probably a better choice, as it has human-like hands (bio-inspired), and thus human-like motor repertoire. However, if the experimental question does not have such requirements, then perhaps it is more beneficial to use a smaller, cheaper, and easier-to-program robot, such as NAO.
